# Intelligent Tensioning Method for Prestressed Cables Based on Digital Twins and Artificial Intelligence

**DOI:** 10.3390/s20247006

**Published:** 2020-12-08

**Authors:** Zhansheng Liu, Guoliang Shi, Anshan Zhang, Chun Huang

**Affiliations:** 1 College of Architecture, Civil and Transportation Engineering, Beijing University of Technology, Beijing 100124, China; shiguoliang@emails.bjut.edu.cn (G.S.); zhanganshan@emails.bjut.edu.cn (A.Z.); chunhuang@bjut.edu.cn (C.H.); 2 The Key Laboratory of Urban Security and Disaster Engineering of the Ministry of Education, Beijing University of Technology, Beijing 100124, China

**Keywords:** digital twin, artificial intelligence, prestressed cable, intelligent tensioning, security assessment, sensing equipment

## Abstract

In this study, to address the problems of multiple dimensions, large scales, complex tension resource scheduling, and strict quality control requirements in the tensioning process of cables in prestressed steel structures, the technical characteristics of digital twins (DTs) and artificial intelligence (AI) are analyzed. An intelligent tensioning of prestressed cables method driven by the integration of DTs and AI is proposed. Based on the current research status of cable tensioning and DTs, combined with the goal of intelligent tensioning, a fusion mechanism for DTs and AI is established and their integration to drive intelligent tensioning of prestressed cables technology is analyzed. In addition, the key issues involved in the construction of an intelligent control center driven by the integration of DTs and AI are discussed. By considering the construction elements of space and time dimensions, the tensioning process is controlled at multiple levels, thereby realizing the intelligent tensioning of prestressed cables. Driven by intelligent tensioning methods, the safety performance evaluation of the intelligent tensioning process is analyzed. Combined with sensing equipment and intelligent algorithms, a high-fidelity twin model and three-dimensional integrated data model are constructed to realize closed-loop control of the intelligent tensioning safety evaluation. Through the study of digital twins and artificial intelligence fusion to drive the intelligent tensioning method for prestressed cables, this study focuses on the analysis of the intelligent evaluation of safety performance. This study provides a reference for fusion applications with DTs and AI in intelligent tensioning of prestressed cables.

## 1. Introduction

At present, intelligent development strategies are being combined to promote the transformation and upgrading of the construction industry to intelligent construction [1], thereby subverting the traditional design–construction–operation and maintenance monitoring model and realizing the design–virtual interaction–intelligent construction–intelligent monitoring of the new model. While the construction industry is transforming and upgrading, large-span spatial structures have also attracted increasing attention, particularly in stadiums. The construction of large-span spatial structures is an important standard for evaluating the construction technology and level of a country. Spatial structures and their construction process have the characteristics of long periods, wide cross-disciplines (design, construction, monitoring, operation, and maintenance), multi-subjects, and strict quality control, resulting in complex construction processes and many abnormal disturbance factors. Thus, the decision-making control method based on manual experience in the traditional construction mode can no longer meet the current development requirements for intelligent space structure construction.

In a large-span spatial structure, the prestress level directly determines the overall performance of the structure [2], and the prestress construction requires higher precision and safety guarantees. Therefore, cable tension construction has become a focus and challenge of prestressed steel structure construction. Research on tension formation and safety control has also become a hot topic in the field of civil engineering. Chen et al. [3] conducted research on a new type of tension construction forming method, construction error influence, and control technology for cable domes. Ge et al. [4] conducted a simulation analysis of the entire load-bearing process for a cable dome structure to improve the construction accuracy. Basta A. et al. [5] conducted a quantitative evaluation of the deconstruction of cable net structures based on building information models. Goh M. et al. [6] studied the simulation of a modular prestressed steel structure construction process based on lean production theory. Although many experts and scholars have conducted research on cable construction accuracy and error analysis, they have not been able to achieve interaction between the virtual and real construction processes to realize intelligence in the entire construction process.

Digital twins (DTs), as a link between the physical world and the virtual digital space [7], are a key technology for intelligent construction. Artificial intelligence (AI), the internet of things, blockchains, big data, and other technologies are coupled and integrated with DT-related technologies, such as simulations, virtual reality, and twin modeling, thus making it possible to establish a synchronous operation in the digital space. As a result, system integration becomes possible. DT technology has produced obvious application value in the aerospace industry, where it has realized intelligent management and decision-making for the entire life cycle of aviation products and the building of digital clues based on DT technology [8]. A DT is a high-fidelity dynamic virtual model that simulates the state and behavior of physical entities [9]. At present, the application range of DT technology has expanded from the aircraft field to other fields. DT-related technologies mainly involve the application of visualization technologies, such as virtual reality and augmented reality, to realize the dynamic perception of the real physical world and the presentation of virtual digital spaces, thereby achieving transparency in the entire process [10]. Tao et al. [11] designed the system architecture for a DT workshop through research on the characteristics and key technologies of DTs and conducted a systematic exploration to realize virtual–real interaction in twin workshops. Thus, the theoretical basis of the cyber–physical integration and key technologies were summarized to provide a reference for the realization of DT workshops. Bicocchi, N. et al. [12] studied the interoperability architecture of a digital factory and noted that a core aspect of the digital factory was to enable the collaboration of stakeholders in the product life cycle through the use of software solutions. As a result, a combination of a service-oriented and architectural framework of the characteristics of the data sharing system was proposed.

Artificial intelligence (AI) has been applied in many disciplines and formed a variety of intelligent algorithms. Especially with the rapid development of deep learning [13] in artificial intelligence, artificial intelligence has become a research and application hotspot in various fields. Jumani T. A. et al. [14] developed an optimal microgrid controller using the particle swarm optimization algorithm (SSA), which effectively controls carbon dioxide emissions through intelligent analysis of the dynamic response of the power system and reduces the overload of the power system. Javani B. et al. [15] proposed a fast convergence path based on a dynamic traffic allocation algorithm, which provides an idea for the evaluation of intelligent transportation systems in urban planning. Ataei M. et al. [16] developed a performance prediction model of a cutting machine by combining a multi-layer artificial neural network and a genetic algorithm, the support vector regression algorithm, and the cuckoo optimization algorithm, and realized the intelligent evaluation of cutting machine performance. Nilashi M. et al. [17] used deep belief networks (DBN) and support vector regression (SVR) to predict neurological diseases and improved the accuracy and scalability of predictions by establishing data models. Hu et al. [18] proposed a bearing remaining service life prediction model based on a deep belief network (DBN). The proposed method improves the prediction accuracy and ensures the reliability of bearing use. The intelligent algorithms in artificial intelligence provide a platform for data analysis and processing and will play a leading role in the implementation of projects in the engineering field.

Through comparative analysis, the application of DT technology in the manufacturing industry is relatively mature. However, in construction industry applications, the twin model is mostly used in the stage of “reflecting reality” [19]. Thus, achieving “virtual and real interaction, and virtual control of reality” and supporting the “dynamic perception, time-course processing, intelligent diagnosis, and intelligent decision-making” under an intelligent tensioning of prestressed cables mode also require the combination of DT and AI technologies. Through the combination of DTs and AI, particularly intelligent algorithms [20], the entire process of tensioning can be digitalized and made intelligent. On the one hand, the collection of multi-source heterogeneous construction process data fused into the digital space twin model can realize real-time updates of laws and theories through AI technology, in particular deep learning, thereby supporting intelligent diagnoses and scientific prediction. Thus, the accumulation of theoretical rules in the AI model is realized to solve the problem of information blocking in long-term construction processes. On the other hand, in the case of deep integration of AI and DTs, the ability of an AI model to diagnose and predict results through a DT model is simulated and verified in various models in the digital space, providing an efficient and reliable means of trial and error for the results of intelligent diagnosis, thereby improving the correctness and accuracy of cable tensioning.

At present, research on the combined application of DTs and AI technologies is still in its infancy. Based on the tensioning process of prestressed cables, this study explores the fusion mechanism of DTs and AI, and the key technology driving the intelligent tensioning of prestressed cables. Based on sensor equipment and intelligent algorithms, a multi-dimensional and multi-scale twin agent modeling of the cable tensioning system is established, and the elements of the performance optimization of the tensioning system are analyzed to control the tensioning process at multiple levels, thereby realizing the intelligent tensioning of prestressing cables. Driven by the intelligent tensioning method, the safety assessment of the intelligent tensioning process is analyzed. By establishing a high-fidelity twin model and a three-dimensional integrated data model, intelligent closed-loop assessment of safety performance is realized.

## 2. Fusion Mechanism of DT and AI

The essence of the tensioning system driven by the fusion of DTs and AI lies in the realization of simultaneous mapping of a real-world multi-dimensional tensioning system to the virtual space through the twin models of the virtual space (i.e., the geometric model, physical model, behavior model, and rule model) [21]. The visualization and digitization of all elements of the tensioning system through the virtual model simulation reality provide collaborative feedback to ensure they run synchronously with the physical tensioning system, thus realizing a virtual reflection of reality and virtual control of reality. Through the integration of AI, particularly deep learning and other techniques, the intelligence and coordination of the operation and control of the tensioning system can be realized, and intelligent diagnosis and scientific prediction of the data-driven system can be achieved. Eventually, the physical tensioning system in the physical dimension and the virtual tensioning system in the information dimension will be coordinated and controlled through interactive feedback, and a new model of the tensioning system with deep learning and intelligent optimization effects will be constructed.

**Figure 1:**
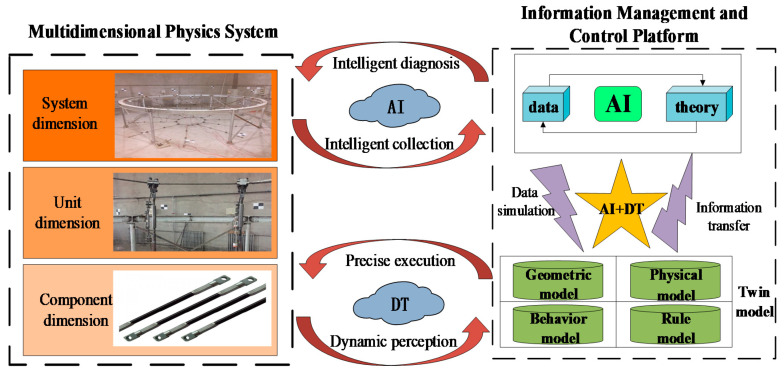
Digital twin (DT) and artificial intelligence (AI) integration mechanism.

As illustrated in Figure 1, A mechanism for the fusion of digital twins and artificial intelligence is built. The basis of this fusion mechanism is the intelligent collection and dynamic perception of the tension information of the real structure. In this study, intelligent sensing devices such as three-dimensional laser scanners and sensors are used to intelligently collect and dynamically perceive information such as the environment and mechanical properties of the structure, thereby providing a basis for the establishment and intelligent analysis of the structural twin model. The information management and control platform based on deep learning is the core of the integration of DT and AI technologies. The intelligent tensioning information management and control platform formed by the fusion of these two technologies mainly consists of two parts: an AI brain and a DT model. This two-part information management and control platform for the efficient operation of the tensioning system is also the core of the intelligent diagnosis and scientific prediction of the intelligent tensioning system. By building an information management and control platform, real-time perception of multi-dimensional and multi-scale tensioning information (from the component dimension and unit dimension to system dimension) can be realized, allowing for accurate analysis and guidance of the tensioning process.

**Figure 2:**
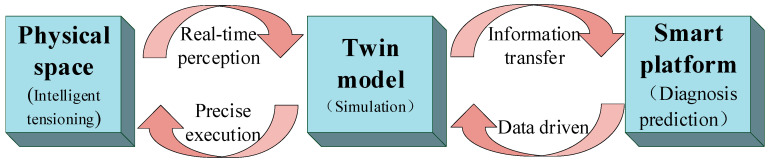
Closed loop formed by the information management and control platform.

The information management and control platform accepts the input of the perceived information of the tensioning system. This becomes the output of the tensioning system control instruction after analysis and processing or is stored in the information management and control platform as theoretical rules. Many intelligent algorithms for different scenarios in tensioning system information management and control platforms have been collected to form the diagnosis and prediction center of the tensioning system. Its main function is to collect, process, and analyze information and then output the diagnosis and decision data for the structure to govern and control the operation of the tensioning system. The important features of the information management and control platform are fusion and integration. Fusion refers to the coordinated consideration of the influencing mechanisms of various functions and the diagnosis of the entire tensioning system. Integration refers to the integration of separate and sub-item processes into a complete construction process, thereby ultimately ensuring the overall integrated operation of the tensioning system. The DT has the function of perception transmission. In the information management and control platform, the behavior information of the tensioning system is transmitted to the AI brain through the virtual–real interaction of the DT. The diagnosis and prediction information produced by the intelligent algorithm is transmitted to the real tensioning structure through the DT system, thus forming a closed loop and realizing the virtual control and precise execution of the tensioning process. The closed loop formed by the information management and control platform is depicted in Figure 2.

The intelligent algorithm model [22] given by the AI technology provides support for the intelligent diagnosis and scientific prediction of the tensioning system, and it is the core of the data analysis and processing performed in the information management and control platform. In this study, driven by a deep belief network (DBN), the environmental effects and structural performance of the structure of the construction completion process are used as historical mining data for the training set of the intelligent algorithm to establish a cable tension analysis algorithm architecture. By substituting real-life collection and simulation data into the training algorithm to predict the future development trend of the tensioning process, we thus establish a three-dimensional integrated data model that integrates historical data, collected and simulated data, and predicting data. The construction process optimization, material resource scheduling, quality prediction, and damage diagnosis of the tensioning system are analyzed and solved through the data model, and the results are imported into the DT model for simulation, thereby providing intelligent diagnosis and scientific prediction support. The channel for data perception and fusion in the virtual digital space is a DT. Collecting real-time behavioral data of the tensioning system in the twin model can reveal the operational details of the real physical world and realize visualization. The rule model in the DT can also “mandatorily” control the tensioning site and deal with the unfavorable scenarios simulated in the behavior model over time [23].

Through analysis of the fusion mechanism of the DT and AI technologies, it can be concluded that the intelligent tensioning system of prestressed cables driven by the fusion of the two has the following characteristics:

(1) Real-time perception is based on virtual reality. Through the information collected by the physical tensioning system and the establishment of a virtual model for the entire tensioning process, visualization of the tensioning process is realized, and full-element, multi-dimensional, multi-scale information of the tensioning system is obtained. Integration provides a synchronous operation model for the construction site.
(2) Data-driven intelligent diagnosis. Through the information management and control platform, the DT model and deep learning are combined, and thus the system can make full use of multiple construction information sources, such as historical data, real-time data collection, and simulation data. In addition, the system can analyze various data, provide intelligent diagnosis of the tensioning process, and allow for timely avoidance of construction risks.
(3) Virtual control and real scientific prediction are realized through AI technology, particularly deep learning and other techniques, to establish data models and intelligent solutions through twin models for simulation. Finally, feedback to the actual tensioning system can be established to achieve precise control of the tensioning process, while the tension safety can be evaluated simultaneously.

## 3. Fusion of DT and AI for Intelligent Tensioning of Prestressed Cables

### 3.1. Multi-Dimensional and Multi-Scale Twin Agent Modeling of the Cable Tensioning System

Through the entire life cycle of the structure, the link between the physical world and virtual reality space is the DT [24]. Based on the DT, the integration and application of AI for data collection, processing, analysis, and mining, followed by simulation and diagnosis of the tensioning process, can improve the correctness and effectiveness of the physical system. In the complex tensioning process for prestressed cables, realizing the fusion drive of DTs and AI is premised on building a multi-dimensional fusion twin agent of the physical space, information space, and business space of the tensioning system.

**Figure 3:**
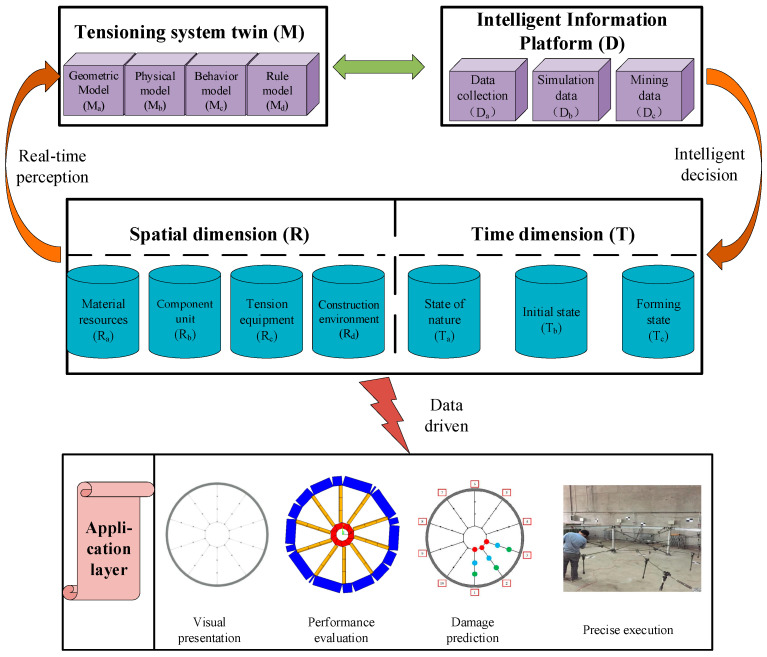
Construction of twin multi-dimensional agents of the tensioning system.

In the context of the development of autonomous intelligence, particularly intelligent body technology, new ideas and methods for modeling intelligent twins of tensioning systems have been developed [25]. Relying on independent intelligent technology, fully considering the temporal and spatial dimensions, the DT technology is integrated in the spatial dimension to build intelligently tensioned material resources, component units, tensioning equipment, and construction environments. This enables the multi-scale longitudinal dimension of the tensioning system modeling to be realized, and the dynamic evolution of the cable tensioning process from the natural state to the initial state and final form [26] is described in the time dimension. A dynamic cooperative operation mechanism based on twin agents is established to support intelligent tensioning, virtual and real interactive configuration modeling, and intelligent tensioning process modeling in multi-dimensional and multi-scale time and space domains. This allows for multi-element, multi-process, and multi-business time-course parallel simulations and virtual–real integrated control of the intelligent tensioning system. The construction of the twin multi-dimensional agent for the tensioning system is depicted in Figure 3.

In the cable tensioning process, a twin model is established based on the data for the spatial and temporal dimensions. The materials, components, equipment, and environment of the spatial dimension are combined with the natural state, initial state, and forming state of the temporal dimension to correspond to the twin model. The geometry, physics, behavior, and rules of the model are used to facilitate interactive feedback of the virtual space to the real world.

The expression for the space dimension is $R={\left(R_{a},R_{b},R_{c},R_{d}\right)}^{T}$; the specific matrix form is

(1) $$R=\left(\begin{array}{c} R_{a}\\ R_{b}\\ \begin{array}{c} R_{c}\\ R_{d} \end{array} \end{array}\right)=\left(\begin{array}{ccc} R_{a1} & R_{a2} & \begin{array}{cc} R_{a3} & \begin{array}{cc} \cdots & R_{\mathrm{am}} \end{array} \end{array}\\ R_{b1} & R_{b2} & \begin{array}{cc} R_{b3} & \begin{array}{cc} \cdots & R_{\mathrm{bm}} \end{array} \end{array}\\ R_{c1} & R_{c2} & \begin{array}{cc} R_{c3} & \begin{array}{cc} \cdots & R_{\mathrm{cm}} \end{array} \end{array}\\ R_{d1} & R_{d2} & \begin{array}{cc} R_{d3} & \begin{array}{cc} \cdots & R_{\mathrm{dm}} \end{array} \end{array} \end{array}\right).$$

In Equation (1), R_a_ refers to the material resources; R_b_ is the component unit; R_c_ represents the tensioning equipment; R_d_ denotes the construction environment; R_a1_, R_a2_, R_a3_, …, R_am_ are the materials at different moments of the construction process; R_b1_, R_b2_, R_b3_, …, R_bm_ represent the components at different moments in the construction process; R_c1_, R_c2_, R_c3_, …, R_cm_ represent the tensioning equipment at different moments in the construction process; and R_d1_, R_d2_, R_d3_, …, R_dm_ represent the construction environment at different moments in the construction process. All the information in the above spatial dimensions is combined with time changes, and the virtual twin modeling is assisted by sensing equipment.

The time dimension is expressed as $T={\left(\begin{array}{ccc} T_{a}, & T_{b}, & T_{c} \end{array}\right)}^{T}$. The specific matrix form is as follows:

(2) $$T=\left(\begin{array}{c} T_{a}\\ T_{b}\\ T_{c} \end{array}\right)=\left(\begin{array}{ccc} T_{a1} & T_{a2} & \begin{array}{cc} T_{a3} & \begin{array}{cc} \cdots & T_{\mathrm{an}} \end{array} \end{array}\\ T_{b1} & T_{b2} & \begin{array}{ccc} T_{b3} & \cdots & T_{\mathrm{bn}} \end{array}\\ T_{c1} & T_{c2} & \begin{array}{ccc} T_{c3} & \cdots & T_{\mathrm{cn}} \end{array} \end{array}\right).$$

In Equation (2), T_a_ represents the natural state; T_b_ represents the initial state; T_c_ represents the forming state; T_a1_, T_a2_, T_a3_, …, T_an_ represent the various construction elements in the natural state; T_b1_, T_b2_, T_b3_, …, T_bn_ represent the various construction elements in the initial state; and T_c1_, T_c2_, T_c3_, …, T_cn_ represent the various construction elements in the forming state. During the construction process, three typical conditions are monitored and various construction elements are integrated to provide an important basis for the establishment of the twin model.

The model matrix established by the integration of the spatial and temporal dimensions is expressed as $M=\left(R,T\right)={\left(\begin{array}{cc} M_{a}, & \begin{array}{cc} M_{b}, & \begin{array}{cc} M_{c}, & M_{d} \end{array} \end{array} \end{array}\right)}^{T}$, where M_a_ is a geometric model, M_b_ is a physical model, M_c_ is a behavioral model, and M_d_ is a rule model. As a result, the modeling of the multi-dimensional and multi-scale virtual twin of the tensioning system is completed, and the established model integrates spatial information with temporal changes, which can realize the virtual and actual interaction and dynamic perception of the tensioning process.

**Figure 4:**
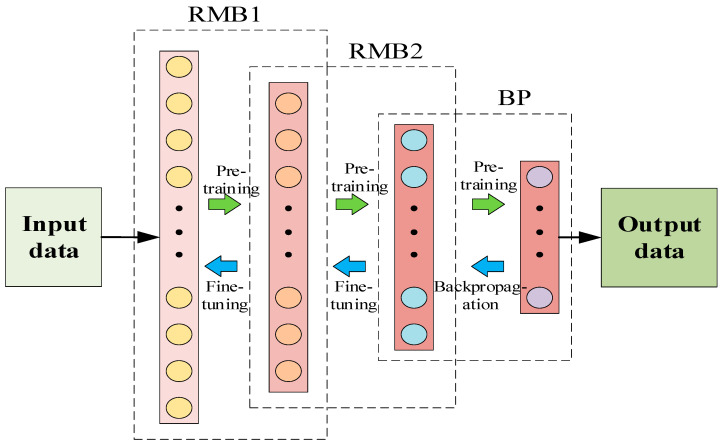
Deep belief network structure diagram.

In addition to establishing the twin model and realizing the intelligent diagnosis and scientific prediction of the tensioning system, a data processing analysis model was also established on the intelligent information platform. In the process of establishing the data model, a deep belief network (DBN) is used to process information. The deep belief network is composed of multiple layers of restricted Boltzmann machines (RBM) and the outermost back propagation network (BP). The structure of the deep belief network established in the security analysis process of this research is shown in Figure 4. DBN information processing is divided into two processes, namely unsupervised pre-training and supervised reverse fine-tuning. In the pre-training, the RBM is trained from bottom to top. After the first RBM is trained, the input of the next RBM is the output of the current RBM, and the training is repeated layer by layer to continuously optimize the parameters of the model to achieve local optimization. In the reverse fine-tuning process, the last layer of the DBN uses the BP algorithm to propagate the training error from high to low to the RBM layer and then fine-tune the DBN architecture to achieve the global optimum [27]. In the cable tensioning process, the entire DBN architecture is trained using historical mining information (information on the procedures that have been completed on the cable) as a sample. The framework completed by the training tests the real-time collected and simulated data to form predicted data for the tensioning process. As a result, a three-dimensional data model integrating historical data, collected and simulated data, and state predicted data is formed. The model is expressed as $D={\left(\begin{array}{ccc} D_{a}, & D_{b}, & D_{c} \end{array}\right)}^{T}$, where the specific matrix form is as follows:

(3) $$D=\left(\begin{array}{c} D_{a}\\ D_{b}\\ D_{c} \end{array}\right)=\left(\begin{array}{ccc} D_{a1} & D_{a2} & \begin{array}{cc} D_{a3} & \begin{array}{cc} \cdots & D_{\mathrm{an}} \end{array} \end{array}\\ D_{b1} & D_{b2} & \begin{array}{ccc} D_{b3} & \cdots & D_{\mathrm{bn}} \end{array}\\ D_{c1} & D_{c2} & \begin{array}{ccc} D_{c3} & \cdots & D_{\mathrm{cn}} \end{array} \end{array}\right).$$

In Equation (3), D_a_ represents the real-time collected and simulated data; D_b_ represents the state predicted data; D_c_ represents the historical mining data; D_a1_, D_a2_, D_a3_, …, D_an_ represent the real-time collected and simulated data for various construction elements; D_b1_, D_b2_, D_b3_, …, D_bn_ represent the state predicted data for various construction elements; and D_c1_, D_c2_, D_c3_, …, D_cn_ represent the historical mining data for various construction elements.

Through the fusion of the DT and AI methods, virtual models and data models are established to build a multi-dimensional and multi-scale twin agent model of the cable tensioning system. This is an important component for realizing dynamic perception, intelligent diagnosis, scientific prediction, and precise execution of cable tensioning.

First, by establishing a DT model of the tensioning system, the different types of twins (geometry, physics, behavior, rules) are defined, and the data interaction capability of the twin model and the real tensioning system is constructed. Through integration of the agent technology under the guidance of the AI technology, the spatial and temporal dimensions of the data are learned, processed, and mined. Under the interactive collaboration of intelligent algorithms and twin models, simulations are performed on many elements of the tensioning process, and the results guide the actual construction, thus achieving visualization of the tensioning process, intelligent diagnosis, scientific prediction, and precise execution.

### 3.2. Fusion of Digital Twins and Artificial Intelligence for Performance Optimization of the Tensioning System

**Figure 5:**
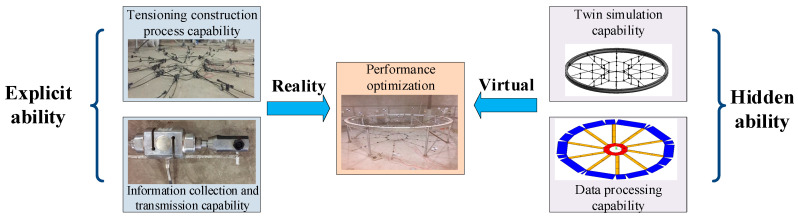
Tensioning system optimization based on twin intelligence.

The purpose of the fusion of DTs and AI for the process of prestressed cable tensioning is to realize the visual presentation, intelligent diagnosis, and scientific prediction of the tensioning system. Through global optimization and control of the cable tensioning system, the accuracy and intelligence of cable tensioning construction can be improved [28]. Based on the realization of multi-dimensional and multi-scale twin agent modeling of the tensioning system, another problem that needs to be solved is the deep integration of the twin model and AI technology to improve the overall optimization and decision-making ability of the tensioning system. The optimization and improvement of the overall performance of the tensioning system depend on explicit capabilities, such as the construction process and information collection that are determined by the tensioning equipment, as well as hidden capabilities, such as the data-driven twin simulation and mining processing, as depicted in Figure 5.

The explicit capabilities are expressed as $E={\left(\begin{array}{cc} E_{a}, & E_{b} \end{array}\right)}^{T}$, where E_a_ is the tensile construction process capability, and E_b_ is the information collection and transmission capability. The hidden capabilities are expressed as $H={\left(\begin{array}{cc} H_{a}, & H_{b} \end{array}\right)}^{T}$, where H_a_ is the twin simulation capability, and H_b_ is the data processing capability. Only continuous improvement of the explicit and hidden capabilities can ensure continuous optimization of the performance of the intelligent tensioning system. The system performance optimization is expressed as P = (E, H).

On the one hand, in the actual tensioning process, the tensioning construction technology and the information collection and transmission capabilities are the explicit abilities required to realize the performance optimization of the intelligent tensioning system. An intelligent twin model of the tensioning equipment and structure can be constructed and integrated with computer image recognition, internet of things, and other transmission technologies. Then, combined with the real-time monitoring data in the actual tensioning process obtained from the sensor equipment, dynamic perception, optimization models, driving analysis, and evaluation of the status of equipment and components in the tensioning process can be performed to realize the time-course management and control of the tensioning process, thereby enhancing the explicit ability of the cable tensioning process.

On the other hand, in the virtual digital space constructed based on the tensioning process, analog simulation and data processing capabilities comprise the hidden capabilities required to achieve performance optimization of the intelligent tensioning system. The construction of an efficient and reliable tensioning construction system depends on the continuous optimization and management of the tensioning system. By establishing a collaborative interaction mechanism based on the tensioning system twin agent in the virtual digital space, DTs and intelligent algorithms can be integrated to analyze and mine the tensioning information; simulate the tensioning process; realize overall prediction, evaluation, and control of the tensioning system; and improve the hidden ability of the tensioning process.

## 4. Tensioning System Control Center Driven by the Integration of DTs and AI

**Figure 6:**
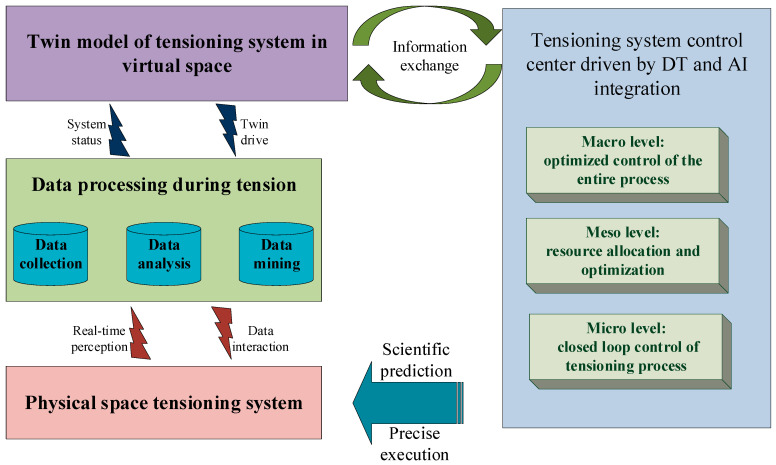
Construction of a tensioning system control center driven by the fusion of DTs and AI.

Prestressed cable tensioning construction is a complex and systematic project with multi-element, cross-professional, strong coupling, and multi-dimensional characteristics, and the tensioning quality is easily disturbed by various uncertain disturbance factors. The operation of the tensioning system also has significant dynamic and nonlinear characteristics [29], which results in cumbersome multi-dimensional and multi-element problems of modeling, optimization, and closed-loop convergence control in large tensioning systems. To construct an intelligent cable body model and analyze the performance optimization of the tensioning system, a tensioning system control center driven by the fusion of DTs and AI was constructed, as depicted in Figure 6. With the AI control center of the cable tensioning system as the core, establishment of the overall coordination, optimization, and closed-loop control architecture of the cable tensioning system is key for the continuous improvement of the ability of the tensioning system in a complex interference environment. It is also an important link for realizing intelligent tensioning of prestressed cables.

The proposed system involves the following main components:

(1) Full-factor data modeling and fusion of the tensioning process. Based on the full production factors of the tensioning system, twin modeling of each element is performed to realize dynamic perception, processing, and analysis of multi-source heterogeneous data throughout the tensioning process. In addition, through real-time collection of global information of the tensioning process; historical data capture and mining; and the establishment of a multi-dimensional and multi-scale tensioning process, dynamic information expressions, and data models to achieve the full step, all the elements of the tensioning process data fusion and drive can be captured.
(2) Closed-loop control of the cable tensioning process. At the micro-control layer of the tensioning system, relying on the DT of the tensioning system, a tensioning system for the virtual and real space is constructed by establishing the interaction and fusion mechanism between the tensioning systems in the real physical world and the virtual digital space. The global perception model of the pulling system can realize closed-loop control of the virtual–real interaction and pulling process.
(3) Allocation and optimization of tensioning process resources. At the intermediate control layer of the tensioning system, through establishment of a cross-professional tensioning capability and a deployment coupling mechanism, a multi-level tensioning system twin component model and a large-scale cross-professional resource coordination and optimization model are constructed. This allows for collaborative optimization of resources in the tensioning process under uncertain and complex conditions.
(4) Full-process optimization management and control of the tensioning system. At the macro decision-making level of the tensioning system, AI is used to drive the overall data mining and knowledge accumulation of the tensioning system, and the overall operating status and trends of the tensioning system are diagnosed and predicted to achieve prestress intelligent evaluation and precise execution of the entire cable tensioning process.

## 5. Safety Performance Evaluation for Intelligent Tensioning of Prestressed Cables

Through analysis of the characteristics of the DT and AI technologies, their integration mechanism is summarized, and a multi-dimensional and multi-scale twin agent model of the cable tensioning system is constructed. After construction of the framework, the ability to support the continuous optimization of the system is analyzed, and the tensioning system control center driven by the integration of DTs and AI is explored. On this basis, a safety performance assessment of cable tensioning is conducted to realize the virtual reflection of reality and the virtual control of reality in the cable tensioning process.

The prestressed cable tensioning process in large-span spatial structures is characterized by high construction accuracy requirements and difficult structural damage control [30]. In the traditional tensioning construction process, the various stages of tensioning, monitoring, and optimization control are independent of one another. Thus, real-time management, control optimization, and deployment of the tensioning process cannot be realized. It is difficult to ensure the accuracy of cable tensioning, and the tensioning safety risk control and degree of intelligence for structural damage assessment are low. Structural quality is a decisive factor in ensuring the safety performance of large-span spatial structures, and it is also the focus of strict control over the entire cable tensioning process [31]. Structural safety assessments are important measures to ensure the quality of spatial structures. Specifically, these comprise the comprehensive consideration of factors, such as construction accuracy during the tensioning process while planning a reasonable tensioning process to achieve the desired tensioning quality of the structure. In addition, when safety problems arise, each construction step and link can be captured, and the causes can be determined to improve the tensioning process and control the tensioning quality. The state of the structure is an important factor affecting the reliability of the structure. The various effects that the structure bears and the performance of the structure itself directly affect the state of the structure. Therefore, when evaluating the safety performance of a tensioned structure, it is particularly important to analyze various factors and structural performance parameters that affect the structure.

**Figure 7:**
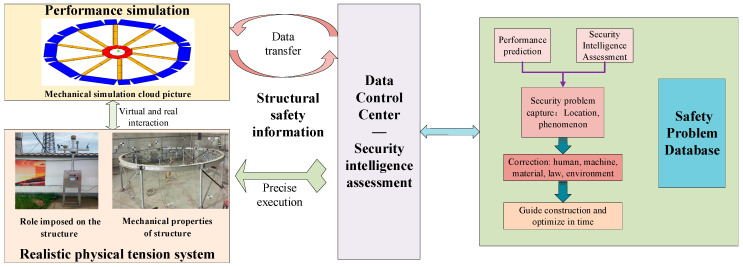
DT and AI fusion drive structure for intelligent safety control.

Intelligent safety control of a structure driven by the fusion of DTs and AI refers to the structural monitoring data based on production elements (human, machine, material, method, ring) and structural monitoring data in the perception cable tensioning system, combined with twin data to drive the structure. The twin model conducts performance simulations and feeds the calculation results back to the safety control module of the data control center to realize integration of the multi-dimensional and multi-source heterogeneous structural safety information, such as tensioning construction sites and simulations [32], as illustrated in Figure 7. In the whole process, it is necessary to conduct real-time collection and analysis of the external effects and structural mechanical parameters of the structure tensioning process, and then intelligently evaluate the safety performance of the structure. In the virtual digital space, real-time information such as the environment and mechanical properties of the real tension structure is collected through a three-dimensional laser scanner and sensor to establish a high-fidelity twin model. By setting the working conditions, the stress, strain, displacement, deflection, and Poisson’s ratio of the structure can be simulated. In this study, three types of working condition information (member length error, temperature effect, load effect) and five types of mechanical parameters (stress, strain, displacement, deflection, Poisson’s ratio) are combined to analyze the cable force by the deep belief network. The safety performance of the structure is evaluated by the cable force change rate. According to the evaluation results, the maintenance is carried out and imported into the twin model for feasibility analysis, then the structure is accurately maintained, and the closed-loop control of the safety evaluation of the tensioning process is realized. Thus, the cable tensioning process and corresponding construction parameters are recorded in the twin database. Through analysis and prediction of the key indicators affecting the structural performance, combined with the experience data and theoretical specifications, the accuracy and intelligence of the tensioning system safety analysis and evaluation can be rapidly captured, accurately executed, and continuously improved by continuously updating the safety problem database.

**Figure 8:**
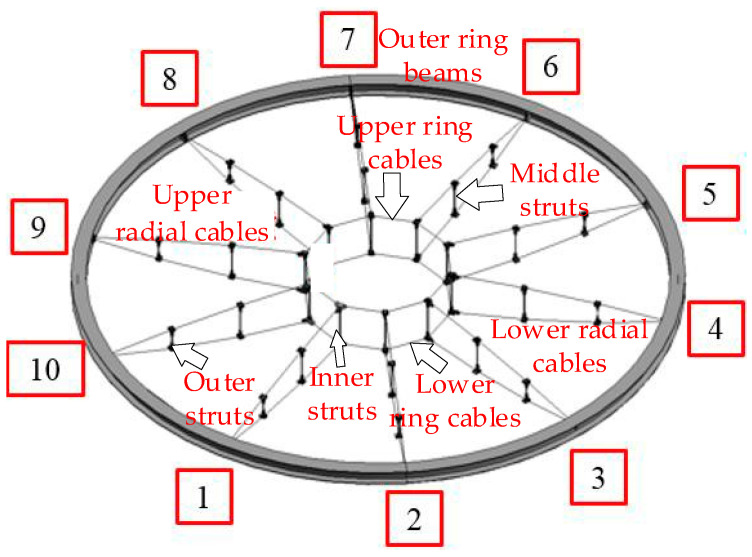
Structural building information modeling model.

Considering a wheel-spoke cable truss as the research object, the intelligent tensioning safety assessment method combining DTs and AI is explored from two aspects: the virtual reflection of reality and the virtual control of reality. The experimental model built in this study is a reduced-scale test model based on a certain wheel-spoke cable truss project. Compared with the actual project, the scale ratio of the test model is 1:10, the cross-sectional area ratio of the cable is 1:100, and the materials are identical. The structure span of the test model is 6 m and consists of 10 radial cables, ring cables, braces, nodes, outer ring beams, and steel columns. The radial cables include upper radial and lower radial cables, and the ring cables include upper ring and lower ring cables. The struts include outer, middle, and inner struts. The structural building information modeling (BIM) model is illustrated in Figure 8.

(1) Reality of the safety assessment

The prerequisite for realizing the intelligent assessment of cable safety during the tensioning process is to ensure the dynamic perception of the virtual and real spaces [33]. In a real tensioning environment, sensors to monitor the structural performance must be arranged first, and the intelligent diagnosis and scientific prediction of the structural safety performance are driven by the data fusion between the measured data of the sensors and the twin model simulation [34]. However, optimizing the placement of sensors is conducive to the later data transmission and processing. The target parameters measured by the sensors can be defined as follows:

(4) $$z=g\left(\begin{array}{cc} x_{1}, & \begin{array}{ccc} x_{2}, & \cdots & ,x_{k} \end{array} \end{array}\right),$$

where x_i_ is the monitoring value directly collected by the sensor on the structure; δ_1_, δ_2_, …, δ_k_ are the errors of x_1_, x_2_, …, x_k_ on the sensor side; and ∆z is the difference in z caused by errors δ_1_, δ_2_, …, δ_k_. Therefore, $z+\Delta z=g\left(\begin{array}{ccc} x_{1}+\delta_{1}, & x_{2}+\delta_{2}, & \begin{array}{cc} \cdots , & x_{k}+\delta_{k} \end{array} \end{array}\right)$. Applying the Taylor expansion, $\Delta z=\frac{\partial g}{\partial x_{1}}\delta_{1}+\frac{\partial g}{\partial x_{2}}\delta_{2}+\cdots +\frac{\partial g}{\partial x_{k}}\delta_{k}$, the maximum error is as follows:

(5) $$\Delta z_{\max}=\pm \left(\left|\frac{\partial g}{\partial x_{1}}\delta_{1}\right|+\left|\frac{\partial g}{\partial x_{2}}\delta_{2}\right|+\cdots +\left|\frac{\partial g}{\partial x_{k}}\delta_{k}\right|\right).$$

**Figure 9:**
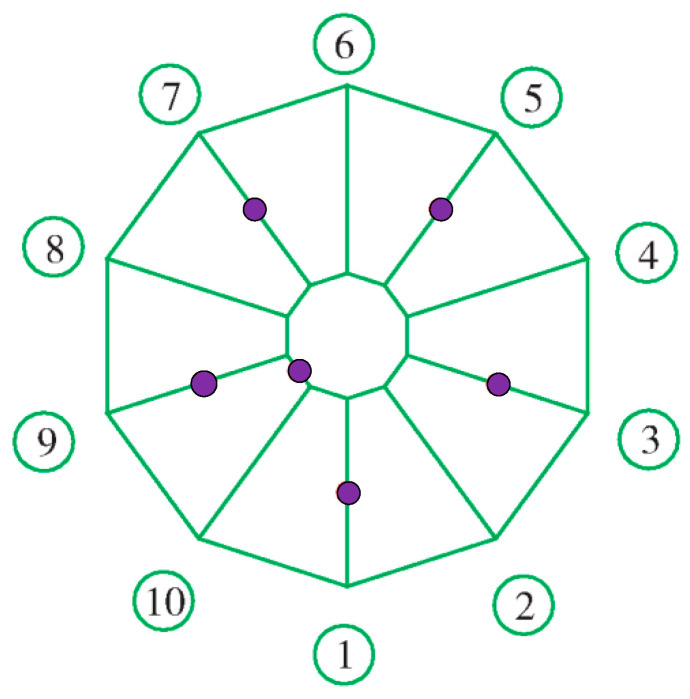
Layout of structural sensors.

From the maximum error, the objective function for the sensor arrangement can be obtained as $Y=\mathrm{Min}(\Delta z_{\max})$. Based on experience with the engineering layout of spatial structure sensors, the sensors are arranged in the key parts of the force and the positions most sensitive to damage [35]. The locations of the structural sensors during the tensioning process in this experiment are illustrated in Figure 9. For the force and strain of the cable, a total of 12 monitoring points are present: 2 monitoring points are arranged for each of the upper and lower ring cables and there are 10 monitoring points for 1, 3, 5, 7, and 9 of the upper and lower radial cables.

In the virtual digital space, the twin model is built using BIM and finite element software. According to the guidelines for establishment of the twin model presented in Section 3.1, the construction of the twin model is driven by fusion of the real-time tensioned spatio-temporal dimension information. The sensor arrangement can collect real-time information on the spatial and temporal dimensions of the tensioning process. The spatial information collected for the tensioning site material resources, component units, tensioning equipment, and construction environment in the spatial dimension is expressed as R = (R_a_, R_b_, R_c_, R_d_)^T^. The information for the three important stages of the tensioning process in the temporal dimension is expressed as T = (T_a_, T_b_, T_c_)^T^. The fusion of the spatial and temporal information drives the establishment of the twin model. The twin model is expressed as M = (M_a_, M_b_, M_c_, M_d_)^T^. By building a high-fidelity twin model to simulate various working conditions in the actual tensioning process, twin data are created for the data measured by the sensor to provide information support for the intelligent diagnosis and scientific prediction of safety assessments. In the process of real-time perception of the actual tension of the BIM model, the structural information of the key construction nodes for the tension is scanned using three-dimensional laser scanning technology, and point cloud data for the tension process are extracted. The time during the tensioning process is established by inverse modeling using a BIM model of spatial intelligent integration, thereby revising the BIM model in the design stage and establishing a geometric model that is consistent with the actual structure.

**Figure 10:**
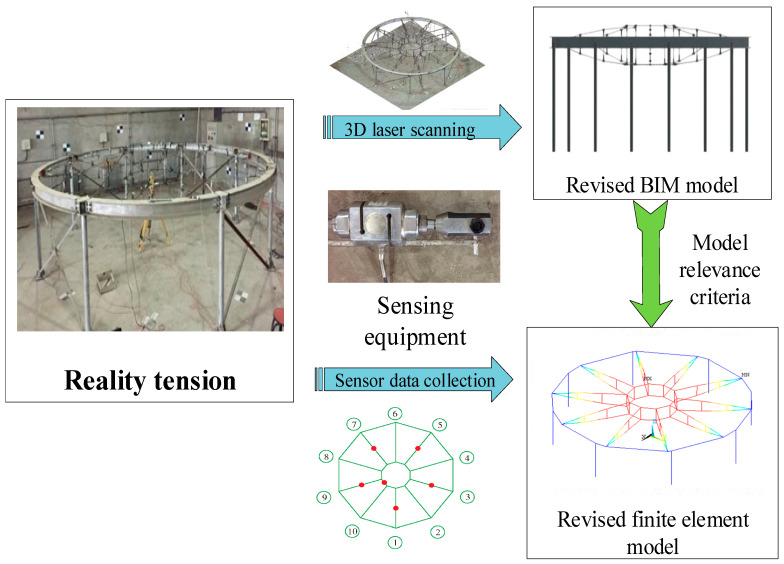
Correction of the twin model.

The finite element model is revised by combining the revised BIM model with the model correlation criterion [36]. The key components and nodes are analyzed in each tensioning construction step, and intelligent fusion of the spatial and temporal dimensions is realized during establishment of the finite element model. In the revision of the model correlation criterion, the cable strain is used as the control index for the fidelity of the twin model, and $\varepsilon_{t}$ is defined as the strain measured by the sensor during the actual tensioning process. The cable strain simulated by the model simulation is $\varepsilon_{m}$, and the fidelity index of the twin model is $e_{\varepsilon }$, where $e_{\varepsilon }=\frac{\left|\varepsilon_{t}-\varepsilon_{m}\right|}{\varepsilon_{t}}\times 100\%$. The revision process of the twin model is depicted in Figure 10. In order to improve the accuracy of simulation, the interactive comparison between the real structure and the twin model is carried out in each construction step. In each construction step, the geometric shape and mechanical parameters of the structure are collected by sensing equipment, and at the same time, simulation data are extracted from the twin model. In this study, every 10% increase in the prestress level during the tensioning process was taken as a construction step; after the tensioning, every 1/8 span load was placed as a construction step. In the twin model, based on the actual tensioning information, the actual tensioning process is realized by setting the working conditions. Considering the cable force as the research object, a comparison of the monitoring capability of the twin model before and after correction under a dead weight load is presented in Table 1.

The comparison of the cable forces before and after model correction indicates that the error of the corrected model is controlled within 3%, achieving high fidelity of the twin model. Driven by smart sensing equipment, the twin model establishment rules intelligently integrate the temporal and spatial dimensions of the tensioning process to establish a twin model that is consistent with the actual tension. As a result, virtual mapping of the geometry, physics, behavior, and rules of the real structure is realized.

(2) Fictitious control of the safety assessment

Based on the establishment of a high-fidelity twin model, twin data are constructed from actual tensioning and model simulations. In the data management and control center, various factors in the tensioning process, structural damage evaluation indicators, and structural resistance parameters are collected, machined, processed, and analyzed [37]. According to the structural reliability analysis, the safety performance of the structure should satisfy R − S ≥ 0, where S is the effect of the structure and R is the resistance of the structure. However, during the tensioning process, the structural performance is easily disturbed by various uncertain disturbance factors. The operation of the tensioning system also has significant dynamic and nonlinear characteristics. Therefore, the effect and resistance of the structure change. The performance evaluation should ensure that R(t) − S(t) ≥ 0. The intelligent control center needs to perform real-time analysis of the entire tensioning process and establish the time-history functions, S(t) and R(t), of the structural action effect and structural resistance, respectively. It is necessary to mine the historical information of the tensioning process, collect and simulate real-time information to obtain future trends, establish a three-dimensional integrated data model D = (D_a_, D_b_, D_c_)^T^, and intelligently predict the safety performance of the structure in real time.

**Figure 11:**
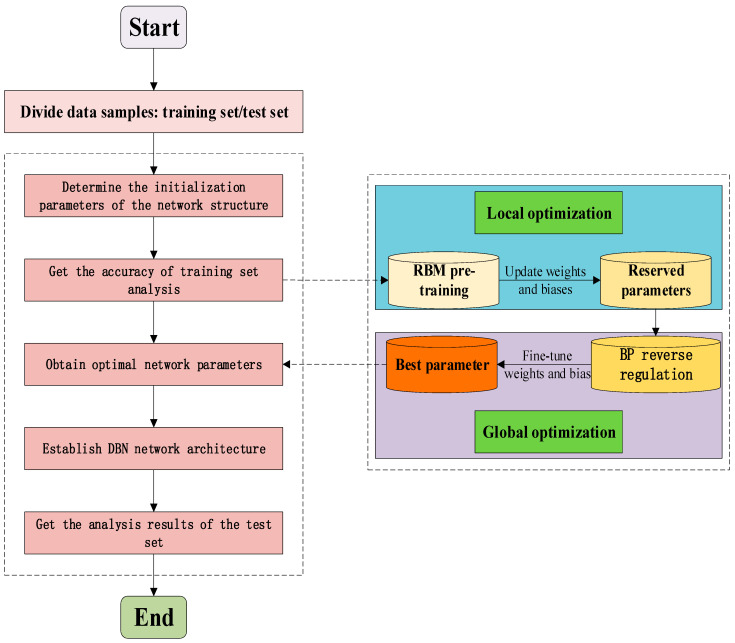
Structural safety performance analysis process.

In this study, because the twin model is corrected for the cable force, the accuracy of other parameters may be insufficient in the simulation of other parameters, so the twin simulation data are analyzed by the deep belief network, and the construction completion process information is used as the training set for structural analysis. From Section 3.1, through the establishment of a suitable framework for the DBN algorithm, the data collected and simulated in real time are used as the test set. The effect of the structure (member length error, temperature effect, load effect) and the mechanical properties of the structure (stress, strain, displacement, deflection, Poisson’s ratio) are used as the input layer of the algorithm, and the cable force is used as the output layer of the algorithm. Finally, the cable force state of the prestressed cable is obtained. In order to visually judge the safety performance of each component, the safety level of the cable force is output according to the quantitative standard of the cable force limit in Table 2, and the cable force is divided into four levels, a, b, c, and d, in the output layer. At the same time, the twin model simulation can quantitatively judge the rate of change of the cable force. Thereby, a data model for safety analysis is established to judge the safety performance of the structure. The process of analyzing structural safety performance by intelligent algorithms is shown in Figure 11.

**Figure 12:**
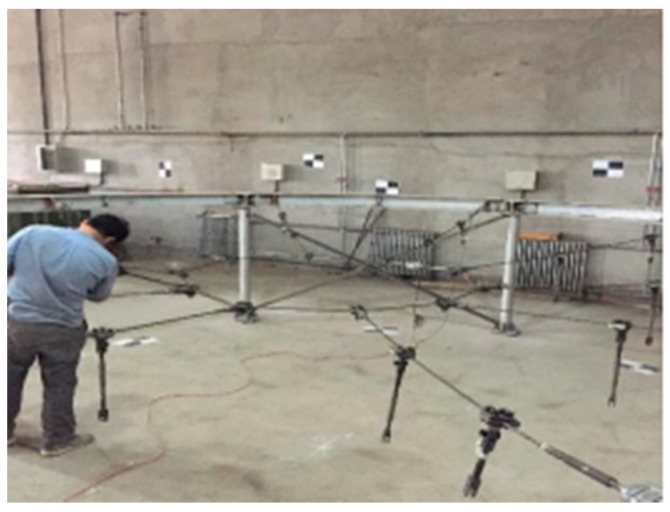
On-site precise maintenance.

During the tensioning process, a high-fidelity twin model and a three-dimensional integrated intelligent data model are used to analyze the changes in the cable force under a quarter-span live load. The safety performance analysis results of the structure are shown in Table 3. It can be seen from Table 3 that the cable forces of the lower radial cable and the lower ring cable vary the most. At the same time, the cable force of the upper ring cable and the upper radial cable has decreased. From this, the main factors affecting the safety performance of the structure are found, and they need to be monitored and maintained. Therefore, with the establishment of the twin and data models, structural safety problems of the tensioning process can be captured, the development of structural safety performance trends can be predicted, and the locations and causes of damage can be accurately determined. The description of the damage problem can be fed back to the twin model to make adjustments to specific construction elements, and the feasibility of maintenance is evaluated in the twin model, thereby realizing virtual control of the intelligent tensioning safety assessment. The precise maintenance of the actual structure based on the intelligent analysis of cable force changes is shown in Figure 12.

In the process of information transmission from the real tension to the twin model, a three-dimensional laser scanner and sensors are used to collect the geometrical shape and mechanical parameters of the structure in real time and combined with the DBN algorithm to intelligently integrate the information of time and space dimensions to establish a twin agent. In the twin model, the real structure is simulated and analyzed, and the data collected by the reality are interactively fed back to realize the one-to-one mapping of the two. The twin agent processes the tension data, updates the simulated physical model in real time, and sends control commands to provide optimization and decision support for physical systems [38]. The DT model can realize simultaneous evolution between the physical world and the digital world, which provides a new technical approach for health monitoring of complex systems. Additionally, the AI technology builds a data model for the evaluation of the structural safety performance, which combines historical mining data, real-time collected and simulated data, and state predicted data to determine the time-history functions of the structural resistance and effect. The integration of DTs and AI can provide technical support for the intelligent assessment of tensioning safety and realize the dynamic perception, intelligent diagnosis, scientific prediction, and precise maintenance of structural damage.

## 6. Discussion and Conclusions

DTs and AI are key technologies for realizing the transformation and upgrading of intelligent tensioning of prestressed cables as well as efficient methods for the management and control of complex tensioning systems. This study analyzes the current status of tensioning construction and the application of information technology, investigates the fusion mechanism of DTs and AI, and introduces the fusion of the two for the tensioning of prestressed cables to realize intelligent tensioning and analysis of the safety performance of intelligent tensioning. This method has the following advantages:

(1) Aiming at the application of DTs at the stage of “reflecting reality with virtual reality,” the integration mechanism of DTs and AI is explored. The combination of the two provides new ideas for the realization of “dynamic perception, real-time processing, intelligent diagnosis, and intelligent decision-making” for intelligent tensioning of the cable.
(2) To apply the fusion mechanism of DTs and AI to cable tensioning, we first perform multi-dimensional and multi-scale modeling to lay the foundation for visualization of the tensioning process, intelligent diagnosis, scientific prediction, and precise execution. Based on the modeling, the ability of deep integration to improve the overall optimized decision-making of the tensioning system is explored.
(3) The construction of prestressed cable tensioning is a complex and systematic project. With the AI control center of the cable tensioning system as the core, a closed-loop control framework is realized to continuously improve the ability of the tensioning system under complex interference environments.
(4) Aiming at evaluation of the safety performance of intelligent tensioning in prestressed cables, intelligent sensing technology and a DBN driven by the fusion of DTs and AI are used to construct a high-fidelity twin model to achieve virtual and real interaction. The integrated data model realizes virtual control of the safety assessment and effectively improves the quality of the tensioning.

In general, the application of DTs and AI to drive intelligent tensioning of prestressed cables is feasible and has broad application prospects. However, current research on intelligent tensioning of prestressed cables is still in the preliminary stages, as is the realization of DTs. By improving the level of information collection and communication technology and real-time analysis of the actual structure, realizing the in-depth integration of digital twins and artificial intelligence and other information technologies to promote the intelligent construction and management of all elements in the entire process of tensioning will be the focus of further research and exploration.

## Figures and Tables

**Table 1:** Comparison of the cable force before and after model correction.

Component Unit Number	Analog Value (N)	Corrected Value (N)	Test Value (N)	Analog Value Error	Corrected Value Error
Upper radial cable 1	5572	4771	4890	14%	−2%
Upper radial cable 3	5563	5711	5850	−5%	−2%
Upper radial cable 5	5570	5083	5150	8%	−1%
Upper radial cable 7	5569	5774	5660	−2%	2%
Upper radial cable 9	5573	5138	5140	8%	0%
Lower radial cable 1	4481	4132	4260	5%	−3%
Lower radial cable 3	4473	4853	4790	−7%	1%
Lower radial cable 5	4569	4132	4100	11%	1%
Lower radial cable 7	4368	4657	4490	−3%	2%
Lower radial cable 9	4481	4303	4210	6%	2%
Upper ring cable	8922	8686	8770	2%	−1%
Lower ring cable	7110	6900	6860	4%	1%

**Table 2:** Quantitative standards of the cable force limit.

Structure Type	Component Category	Ratio of Analysis Value to Design Value			
		a	b	c	d
Cable	Important, secondary components	≥1.00	≥0.93	≥0.90	<0.85
	General components	≥1.00	≥0.91	≥0.86	<0.81

**Table 3:** The safety performance analysis results of the structure.

Component Unit Number	Security Level	Cable Force Change Rate
Upper radial cable 1	b	−4%
Upper radial cable 3	b	−3%
Upper radial cable 5	b	−2%
Upper radial cable 7	b	−3%
Upper radial cable 9	b	−3%
Lower radial cable 1	a	22%
Lower radial cable 3	a	21%
Lower radial cable 5	a	18%
Lower radial cable 7	a	19%
Lower radial cable 9	a	20%
Upper ring cable	b	−2%
Lower ring cable	a	20%
